# Fructooligosaccharides and mannose affect *Clostridium difficile* adhesion and biofilm formation in a concentration-dependent manner

**DOI:** 10.1007/s10096-019-03635-7

**Published:** 2019-07-30

**Authors:** Michał Piotrowski, Dorota Wultańska, Piotr Obuch-Woszczatyński, Hanna Pituch

**Affiliations:** Department of Medical Microbiology, Medical University of Warsaw, Warsaw, Poland

**Keywords:** *Clostridium difficile*, Bacterial adhesion, Biofilm formation, Prebiotics, candidate prebiotics

## Abstract

The aim of this study was to investigate the effects that prebiotic and candidates for prebiotics on *Clostridium difficile* strains to adhere to various human epithelial cell lines and to compare the adhesive properties of specific *C. difficile* strains. We also sought to examine the effect of different concentrations of fructooligosaccharides and mannose on the formation of biofilms by *C. difficile* strains. The influence of cellobiose, fructooligosaccharides, inulin, mannose, and raffinose on the adherence properties of various *C. difficile* strains, including motile 630, non-motile M120, and 10 clinical motile ribotype 027 strains, to non-mucous secreting HT-29, mucous secreting HT-29 MXT, and CCD 841 CoN cells lines. The most effective prebiotics were used in biofilm formation assays. We demonstrated that all *C. difficile* strains adhered to all cell lines. However, the *C. difficile* M120 non-motile strain was statistically more likely to adhere to all three cell lines (CFU median, 40) compared to the motile strains (CFU median, 3; *p* < 0.001). Furthermore, among the carbohydrates examined, only fructooligosaccharides and mannose were found to significantly decrease adhesion (*p* < 0.001) of *C. difficile* strains. Alternatively, using a biofilm assay, we observed, via confocal laser scanning microscopy, that sub-inhibitory concentrations (1%) of fructooligosaccharides and mannose functioned to increase biofilm formation by *C. difficile*. We demonstrated that specific prebiotics and candidate prebiotics exhibit varying anti-adhesive properties towards *C. difficile* in vitro and that treatment with sub-inhibitory concentrations of prebiotics can cause an increase in biofilm formation by *C. difficile.*

## Introduction

*Clostridium difficile* (*C. difficile*) is a Gram-positive anaerobic spore-forming bacterium; it is the primary cause of nosocomial diarrhoea, associated with disturbance of the intestinal microbiota. This microorganism is an etiological agent for antibiotic-associated diarrhoea as well as several clinical complications, including pseudomembranous colitis, toxic megacolon, and intestinal perforation, which has a high mortality rate [1]. The main virulent factors of *C. difficile* are two toxins: toxin A (TcdA; 308 kDa) and toxin B (TcdB; 270 kDa). An additional binary toxin-CDT (CDTa, 48 kDa and CDTb, 74 kDa) is produced by some strains [1, 2].

The hypervirulent epidemic strains (A^+^B^+^CDT^+^) are classified as PCR-ribotype 027/North American Pulsotype 1 (NAP1)/restriction endonuclease analysis (REA) BI type (27/NAP1/BI) toxinotype III and are primarily associated with hospital acquired *C. difficile* infections (CDI) [2]. Specifically, PCR-ribotype 027 (RT027) strains are defined as hypervirulent due to their ability to produce high levels of toxins (A and B) together with their high sporulation capacity and antibiotic resistance [2–4]. Thus, patients infected with RT027 are at twice the risk of succumbing to their infection or of developing a severe CDI compared to patients infected with other strains [5]. In a study that examined the causative agents of CDI in hospitals across Poland (2012–2013), RT027 strains were identified as the most prevalent PCR-ribotype [6].

For many pathogens, the capacity to adhere to host tissues is essential for achieving the first stage of pathogenesis*.* If *C. difficile* is to successfully colonize the gut, it must first access the epithelial cells, which are coated with a mucus layer. This is accomplished through direct adherence to the mucus [7].

Specific cell lines including Caco-2 and HT-29, which have been isolated from colon adenocarcinomas, are commonly employed for in vitro studies examining the attachment of bacterial species. HT-29 cells are characterized as having a small proportion of mucus-secreting cells and columnar absorptive cells, while the mucus-secreting HT29-MTX cells are derived from HT-29 cultures following treatment with methotrexate [8].

Prevention of bacterial adhesion during the early stages of infection can serve to prevent disease development and biofilm formation. Studies have shown that receptor analogs function as efficient anti-adhesive agents and would, thus, be effective primarily against pathogens that bind to human cells. Carbohydrates generally function as efficient cellular receptors and contain similar structures to the glycoproteins or glycolipids for the bacterial adhesins, thereby acting as competitive inhibitors [9]. Prebiotics, which are often saccharides, have thus been described as potential candidates for anti-adhesive therapy. Current definition of a prebiotic is “a substrate that is selectively utilized by host microorganisms conferring a health benefit” [10]. Inulin (INU) and fructooligosaccharides (FOS) are popular oligosaccharide prebiotics that naturally occur in many foods including artichokes, asparagus, leeks, chicory, and garlic [11]. Inulin is a fructan-type polysaccharide carbohydrate, while FOS is a subgroup of inulin, made up of polymers with a degree of polymerization (DP) ≤ 10. INU and FOS are not digested in the upper gastrointestinal tract and, thus, reach the distal portion of the colon in their full form [12]. Raffinose (RAF) is a trisaccharide made up of galactose, glucose, and fructose, which serves as a functional oligosaccharide, and has applications in medicine and food [13]. Several plants sources such as seeds of soy beans, sugar beets and artichoke (Japanese) are rich in raffinose [13]. Raffinose is considered as candidate prebiotics [10, 13]. Cellobiose is a disaccharide that is not digested in the human upper gastrointestinal tract. In the presence of human faecal bacteria, cellobiose was observed to significantly increase production of short-chain fatty acids (SCFA) [14]. d-mannose (MAN) is a monosaccharide that has also been reported to exhibit prebiotic activity and may be beneficial for preventing gut dysbiosis by regulating the balance between harmful and commensal bacteria [15, 16].

The primary aim of this study was to investigate the effects that specific carbohydrates with prebiotic status and candidates for prebiotics chosen based on their varying degree of polymerization have on the ability of motile and non-motile *C. difficile* strains to adhere to various human epithelial cell lines and to compare the adhesive properties of specific *C. difficile* strains to these different cell lines. We also sought to examine the effect of different concentrations of fructooligosaccharides (FOS) and mannose (MAN) on the formation of biofilms by *C. difficile* strains.

## Materials and methods

### Preparation of *Clostridium difficile* cultures and inoculum

Twelve *C. difficile* strains were used in this study. Among them were 2 control strains, *C. difficile* 630 characterized as ribotype 012 (RT012) and an epidemic strain isolated in 1985 from Zurich, Switzerland, *C. difficile* M120 (RT078), and 10 tested strains that were all clinical isolates and toxigenic belonging to the PCR-ribotype (RT027). These strains were isolated from symptomatic patients across Poland [17, 18]. All *C. difficile* strains were collected in the Anaerobic Laboratory, in the Department of Medical Microbiology, at the Medical University of Warsaw. *C. difficile* strains were stored at − 70 °C in a Microbank™ bacterial storage system (Pro-Lab Diagnostics, UK). The strains were thawed before use in experiments, cultured on Columbia Agar plates with 5% sheep blood (Beckton Dickinson, USA), and incubated at 37 °C for 48 h under anaerobic conditions. Clinical isolates were confirmed as *C. difficile* via mass spectrometry (Vitek MS bioMérieux, France). RTs were determined using methods as described by Stubbs et al. [19]. Inoculums were prepared with suspension colonies of *C. difficile* cultured at 37 °C for 48 h under anaerobic conditions on Columbia Agar with 5% sheep blood (bioMérieux, France) and adjusted to a 3.0 McFarland standard.

### Motility assay

Motility assays were performed using motility agar tubes containing brain heart infusion (BHI; Difco, USA) medium (0.175% agar) [20]. The media was inoculated by stabbing with one colony of *C. difficile* that had been previously cultured on Columbia Agar with 5% sheep blood. Results from clinical isolates were compared to those of control strains.

### Prebiotics preparation

Cellobiose (CEL), fructooligosaccharides (FOS), inulin (INU) (from chicory), mannose (MAN), and raffinose (RAF) were purchased from Sigma-Aldrich (USA). Stock solutions (10% *w*/*v*) were prepared using deionized sterile water, microfiltered using a syringe filter (0.2 μm, Corning, USA), and stored at 4 °C.

### Cell cultures

Three human epithelial cell lines were employed throughout the study, namely, HT-29 which are phenotypically non-mucus-secreting cells and were passaged 15–25 prior to use (from the cell-line library at the Anaerobe Laboratory, Department of Medical Microbiology), mucus-secreting HT-29 MTX, passaged 5–15 times (European Collection of Authenticated Cell Cultures, ECACC, UK) and *Homo sapiens* normal colon CCD 841 CoN cells, passaged 5–15 times (American Type Culture Collection, ATCC, USA). Cells were stored in liquid nitrogen at − 196 °C. Cells were cultured in 25 mM 4-(2-hydroxyethyl)-1-piperazineethanesulfonic acid (HEPES) Dulbecco’s modified Eagle medium (DMEM; Life Technologies, UK) with high glucose (4.5 g/L d-glucose), l-glutamine (4.0 mM), supplemented with 10% heat inactivated (30 min at 56 °C) fetal bovine serum (FBS) (Thermo Scientific, USA), and 1% antibacterial solution (Life Technologies, USA) containing 10,000 μg/mL streptomycin, 10,000 U/mL penicillin, and 0.1% amphotericin B (250 μg/mL). All cells were maintained in 75-cm^2^ flasks (Corning, USA) and incubated at 37 °C with 5% CO_2_ and 95% relative humidity. Media was changed every two days. After reaching 100% confluence, cells (the surface is completely covered by the cells) were washed with 10 mL warm (37 °C) phosphate buffered saline (PBS; Thermo Fisher, USA) and harvested with 3 mL of 0.25% trypsin-ethylenediaminetetraacetic acid (EDTA; Sigma-Aldrich, USA) for 5 min at 37 °C. Trypsin was deactivated by adding 10 mL of fresh DMEM with 10% FBS, followed by centrifugation at 1500×*g* for 5 min. Supernatant was discarded, and the pelleted cells were resuspended in 1 mL of fresh DMEM. Cell counts was performed using a Thoma chamber and seeded onto 24-well plates (Corning, USA) at a concentration of 10^4^ cells per well, or re-cultured in a new sterile flask. Cells were observed daily and examined for growth and contamination using an inverted microscope (PZO, Poland). Media without antibiotics and antimycotic substances was used for the last media change. Experiments were performed on mature cells, which was 15 days after seeding HT-29 and CCD 841 CoN cells and 21 days after seeding HT-29 MXT cells [21, 22].

### Adhesion of *C. difficile* strains to human epithelial cell lines

The method employed for determining adhesive properties of *C. difficile* was described previously by Altamimi et al. with specific modifications [22]. All cell lines were prepared as described above. After reaching sub-confluence (70%–80%), cells in 24-well plates were washed twice with PBS, and 400 μL of fresh pre-warmed (37 °C) DMEM without antibiotic/antimycotic solution and with a 1% final saccharide concentration was then added. Medium without saccharides was employed as a negative control. Prepared plates were incubated for 4 h under the abovementioned cell culture conditions. Afterwards, 100 μL of bacterial inoculum was prepared and added to each well and incubated for 1 h. Medium was then aspirated, and wells were washed twice with PBS. The cells were trypsinized for 10 min at 37 °C and 500 μL of fresh media with 10% FBS was added to deactivate the trypsin. The contents of each well were transferred to sterile Eppendorf tubes and diluted 10 times using PBS, and 20 μL was then used to inoculate Columbia Agar with 5% sheep blood. The plates were incubated for 48 h at 37 °C under anaerobic conditions. Every dilution was seeded in duplicate and each assay was performed in triplicate. Colonies were counted and averaged, adhesion percentage was calculated using the formula below (where control represented 100% adhesion) [22].

$$ \mathrm{Adhesion}\ \left(\%\right)=\frac{\mathrm{bacterial}\ \mathrm{count}\ \mathrm{sample}}{\mathrm{bacterial}\ \mathrm{count}\ \mathrm{control}}\times 100 $$

### Biofilm growth and influence of prebiotics on biofilm formation

The most anti-adhesively effective prebiotics FOS and MAN were used in biofilm formation assays. BHI media with different concentrations of saccharides/prebiotics (1%, 2%, 4%, 8%) was pipetted into each well of 96-well flat-bottom microplates (Nunc, Denmark). Three wells for each strain were subsequently inoculated with 20 μL of *C. difficile* culture. Wells containing BHI broth without inoculum were used as negative controls, while positive controls consisted of inoculated wells without prebiotic treatment. Plates were incubated at 37 °C for 48 h under anaerobic conditions. After 48 h, the liquid phase of each well was aspirated using sterile pipettes, washed twice with PBS to remove unattached cells, and air dried at 37 °C for 15 min. Each well was then stained with 1% crystal violet (CV; Chempur, Poland) for 10 min. The CV was removed, wells were washed 8 times with PBS, and air dried for 15 min at 37 °C. The stain was dissolved with 96% ethanol (Hurt-Chem, Poland), and absorbance was measured at A_620_ (Bio-Rad 550 Microplate Reader, Bio-Rad, USA).

### Confocal laser scanning microscopy

Specimens were visualized via confocal laser scanning microscopy (CLSM) according to methods previously described by Waack et al. with modifications [23]. Biofilms were grown on sterile 10-mm-diameter glass bottom dishes (Nunc, Denmark). Overnight cultures of *C. difficile* were diluted in fresh BHI with or without prebiotics. To the experimental conditions, 1% and 8% concentrations of FOS and MAN were added. At 1%, FOS and MAN were seen to induce biofilm growth; while at 8%, both prebiotics effectively inhibited *C. difficile* biofilm formation. Biofilms were allowed to grow for 48 h at 37 °C under anaerobic conditions. Mature biofilms were washed twice using 10 mM MgSO_4_. Biofilms were then stained with acridine orange (10 μg/mL) for 30 min in the dark. Dishes were washed twice with 10 mM MgSO_4_. Imaging was performed using a Nikon A1R MP microscope with a Nikon Ti Eclipse series (Nikon, Japan) under × 60 objective lens using immersion oil. Images were acquired at 2040 × 2048 pixels using a Z-step of 0.1 μm. Acridine orange was detected using an excitation wavelength of 488 nm and emission wavelength of 500–550 nm. Images were processed and analysed with NIS-Elements AR v. 4.10 software.

### Statistical analysis

Statistical analysis was performed using Statistica software (version 13, StatSoft, Poland). Normal distribution of values was confirmed using Shapiro-Wilk test. Differences in adhesion between motile and non-motile strains were evaluated by Mann-Whitney *U* test. The effect of prebiotics on *C. difficile* adhesion was calculated using Kruskal-Wallis one-way analysis of variance followed by Dunn’s test for comparison. Differences in biofilm formation were calculated via one-way analysis of variance (ANOVA) followed by Tukey’s post-hoc test.

## Results

Our study assessed the adhesive capacity of motile and non-motile *C. difficile*, the effects of 5 prebiotics on this adhesive effect, and the influence of chosen prebiotics (FOS and MAN) on biofilm formation.

### *C. difficile* adhesion to human epithelial cell lines

**Fig. 1:**
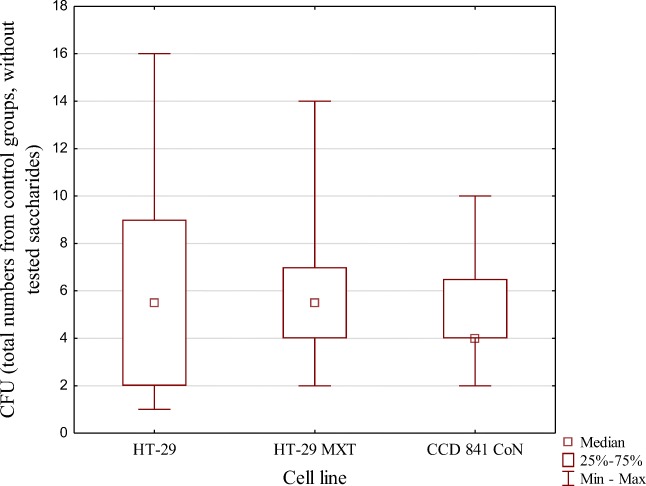
Median adhesion of *C. difficile* strains to the three cell lines

We compared adhesive properties of 10 strains of *C. difficile* RT027 to three human epithelial cell lines, without prebiotics. Our results showed that the specific cell line did not significantly affect adhesion (*p* = 0.65), with a median CFU of 5.5 for HT-29 and HT-29 MXT and 4.5 for CCD 841 CoN cells (Fig. 1).

### Influence of motility on adhesive properties of *C. difficile*

**Fig. 2:**
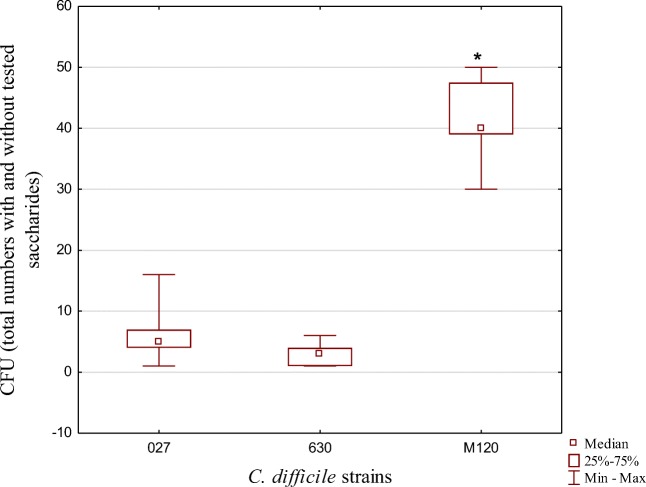
Adhesion of motile clinical *C. difficile* strains belonging to RT027 and two control strains non-motile M120 and motile 630

We next compared the adhesive properties of different *C. difficile* strains with different motility properties. These strains included a motile *C. difficile* 630 strain, a non-motile *C. difficile* M120 strain, and 10 motile clinical RT207 strains. The number of CFUs was compared to those of control groups. We observed a strong, statistically significant correlation (*p* < 0.001) between the number of CFUs and strain motility. The non-motile strain was found to be more likely to adhere (CFU median, 40) to all three cell lines compared to the motile 630 strain (CFU median, 3) or the clinical RT027 strains (CFU median, 5). These relationships are presented in Fig. 2.

### Effect of prebiotics on adhesive properties of *C. difficile*

**Fig. 3:**
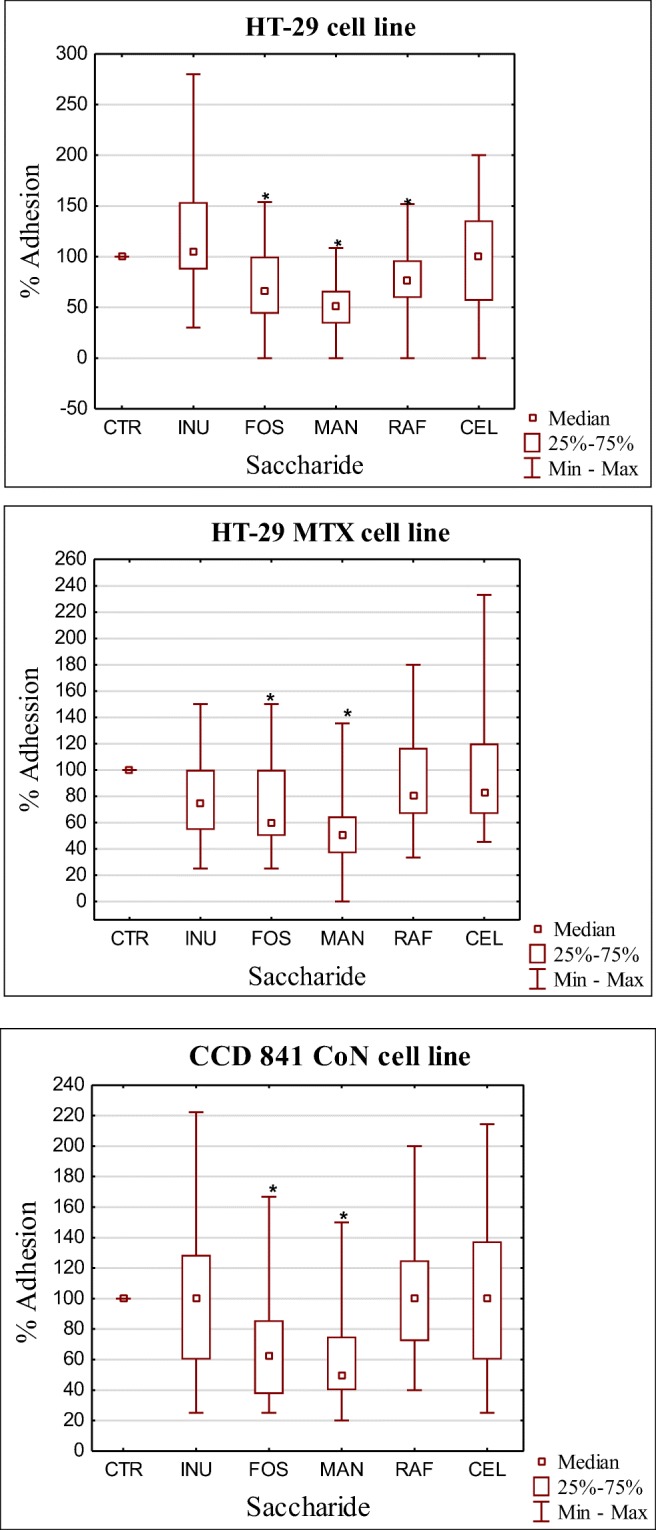
Effect of examined prebiotics on adhesion of *C. difficile* to three cell lines

The primary aim of this study was to examine the effect of 5 prebiotics on the adhesion of 12 *C. difficile* strains (two control strains and 10 clinical strains) to three human epithelial cell lines. FOS and MAN were found to significantly interfere with efficient bacterial adhesion in all cell lines (*p* < 0.001), while RAF only interfered with the adhesive properties of *C. difficile* to the non-mucus-secreting HT-29 cell line (*p* = 0.008). Moreover, CEL and INU were determined to be less effective at inhibiting *C. difficile* adhesion in all cell lines (*p* > 0.05) (Table 1 and Fig. 3).

**Table 1:** Effect of tested prebiotics on *C. difficile* adhesion to the three cell lines

Strain	630	M120	1	2	3	4	5	6	7	8	9	10
*HT-29* (CFU ± SD)												
CTR	5.8 (0.8)	44.5 (3.9)	7.6 (1.0)	6 (0.23)	11.3 (1.2)	5.2 (0.81)	1.2 (0.23)	1.2 (0.23)	1.5 (0.5)	6.7 (2.4)	13 (2.16)	2.2 (0.41)
CEL	2.9 (1.0)	44.3 (5.54)	7.5 (1.47)	7.8 (1.02)	10.8 (1.4)	5.3 (0.41)	1.7 (0.31)	1.5 (0,14)	1 (0.32)	4 (0.62)	14 (1.47)	1 (0)
FOS	4.3 (0.94)	60 (3.55)	5.3 (0.84)	4.7 (0.85)	5.2 (0.62)	2.7 (0.23)	0 (0)	1.2 (0.21)	1.2 (0.21)	2.3 (0.6)	7.2 (0.84)	1 (0)
INU	4.2 (0.94)	44.8 (2.77)	8 (1.02)	7.8 (2.23)	11.8 (1.02)	3.3 (0.62)	2.3 (0.81)	1 (0)	1 (0)	5 (1.21)	13.5 (2.67)	0.83 (0.23)
MAN	2.9 (0.47)	18 (1.64)	7.5 (0.33)	4 (1.0)	12 (1.47)	4.8 (0.96)	0.8 (0.14)	0.4 (0.2)	0.5 (0.11)	4.3 (0.43)	6.3 (1.2)	1.1 (0.11)
RAF	3.1 (0.62)	35.8 (2.25)	10 (1.2)	6.7 (1.1)	11 (1.31)	5 (0.64)	0.8 (0.24)	1 (0)	1.5 (0.35)	5.3 (0.8)	7.2 (0.77)	2 (0.51)
*HT-29 MXT* (CFU ± SD)												
CTR	3 (0.81)	73.3 (4.33)	3 (0.23)	5.7 (0.47)	12.3 (0.77)	7.3 (0.47)	3 (0)	5 (0.69)	4.3 (0.47)	4 (0)	7 (1.69)	3 (0.81)
CEL	3.3 (0.62)	69 (10.5)	2 (0.81)	5 (1.2)	21 (2.11)	7 (1.22)	7 (0)	4.3 (0.61)	3.3 (1.2)	2.7 (0.47)	6.7 (2.49)	2.3 (0.47)
FOS	2 (0)	49 (2.66)	2 (0.81)	2.7 (0.47)	14 (2.11)	9.3 (2.71)	6.3 (0.47)	2.3 (0.47)	2 (0.21)	1.3 (0.35)	10 (2.43)	2.7 (0.94)
INU	3 (0.21)	61 (3.41)	2.6 (0.47)	4.3 (0.47)	11 (2.33)	12.3 (1,93)	3 (0)	4.7 (1.4)	2 (0.47)	2.7 (0.47)	8 (1.69)	1.3 (0.47)
MAN	1 (0)	47.3 (3.55)	2.3 (0.47)	2.7 (0.47)	13 (3.1)	4.3 (0.47)	3 (0.47)	1 (0)	1.3 (0.47)	2 (0.47)	3 (0.47)	1.3 (0.47)
RAF	2 (0.47)	58 (2.36)	2.7 (0.47)	3.3 (1.24)	17.3 (1.41)	10 (1.63)	3.3 (0.47)	1.7 (0.47)	3 (0.47)	3.7 (0.34)	8 (0.94)	3.3 (0.47)
*CCD 841* (CFU ± SD)												
CTR	1 (0)	23 (3.29)	6.7 (0.47)	4.8 (1.11)	10 (0.47)	4.5 (0.62)	4 (0)	5.3 (0.4)	4 (0.81)	4.2 (0.47)	2.3 (0.47)	5.3 (1.24)
CEL	2.2 (0.11)	11.3 (2.16)	13 (1.41)	4.6 (0.91)	7.7 (1.6)	5.6 (0.62)	2.5 (0.47)	5.7 (0.47)	4.3 (0.81)	3 (0)	3 (0)	4.5 (0.71)
FOS	1.3 (0.21)	10 (1.63)	6 (0.94)	2 (0)	3.6 (1.17)	2.2 (0.23)	2 (0.7)	3 (0)	4 (0.41)	2.3 (0.47)	2.5 (1.0)	3.3 (0.62)
INU	1 (0)	7 (0.44)	4.3 (0.94)	1 (0)	17 (2.16)	2.5 (0.23)	2.1 (0.4)	3.6 (0.47)	3.3 (0.4)	1.8 (0.33)	2.5 (0.4)	2.3 (0.47)
MAN	2.2 (0.62)	7.2 (2.09)	7.7 (0.47)	2.7 (0.47)	3.8 (0.47)	2.2 (0.31)	1.7 (0.31)	3.5 (0.23)	2.7 (0.23)	1.6 (0.6)	3 (0)	3.5 (1.08)
RAF	1.3 (0.47)	12.3 (2.3)	12 (1.7)	3 (0.41)	8.8 (0.81)	4 (0)	4.2 (0.47)	3.7 (0.47)	6 (1.64)	3.3 (0.31)	3 (0)	3.8 (1.24)

*CFU* colony forming unit, mean from 6 measurements, *CTR* control (medium without saccharide), *CEL* cellobiose, *FOS* fructooligosaccharides, *INU* inulin, *MAN* mannose, *RAF* raffinose, *SD* standard deviation

### Influence of FOS and MAN on *C. difficile* biofilm formation

To determine the effect of prebiotics on biofilm formation, we only included those that elicited consistent inhibitory effects on the adhesion of *C. difficile* in the previous experiment, namely FOS and MAN. All 12 strains of bacteria were included in this study. Our results revealed that the *C. difficile* M120 strain possessed the strongest ability to form biofilm in vitro with a mean absorbance (A620 nm) from three measurements of 1.93. Among the clinical strains, the highest amount of biofilm was produced by strains 2 (mean A620, 0.80), 8 (mean A620, 0.77), 6 (mean A620, 0.75), and 9 (mean A620, 0.70). Alternatively, the least amount of biofilm was produced by strain 5 (mean A620, 0.33).

**Fig. 4:**
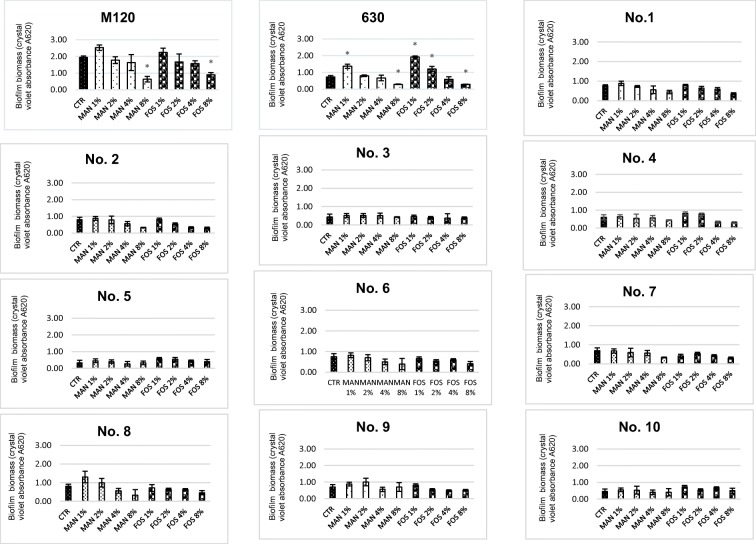
Average biofilm formation by examined *C. difficile* strains with different concentrations FOS and MAN

We examined the effects of the prebiotics at concentrations of 1%, 2%, 4%, and 8%. At 1%, both MAN and FOS acted to induce biofilm growth for all *C. difficile* strains. However, statistically significant differences were observed for strain 630 (MAN 1% *p* < 0.001; FOS 1% *p* < 0.001). In addition, treatment with 2% FOS was found to induce biofilm growth of *C. difficile* 630 (*p* = 0.014). However, all other treatments with prebiotics added at concentrations of 2% and 4% did not significantly affect biofilm formation. When added at a concentration of 8%, MAN acted to significantly reduce biofilm formation by *C. difficile* 630 (*p* = 0.02) and M120 (*p* = 0.004). Similar observations were made for 8% FOS with these two strains of bacteria (*p* = 0.025 and *p* = 0.028, respectively). However, treatment of clinical RT027 strains with MAN and FOS did not cause significant differences in biofilm formation. However, inhibition were observed in strain no 2 following treatment with 8% MAN (*p* = 0.30) and 8% FOS (*p* = 0.22), in strain no 7 by 8% MAN (*p* = 0.18) and 8% FOS (*p* = 0.14), in strain no 8 by 8% MAN (*p* = 0.24) and 8% FOS (*p* = 0.34), and in strain no 4 by 8% FOS (*p* = 0.18). We, therefore, categorized prebiotic concentrations of 8% as inhibitory, and lower concentrations (1%, 2%, 4%) as sub-inhibitory (Fig. 4).

### Confocal laser scanning microscopy

**Fig. 5:**
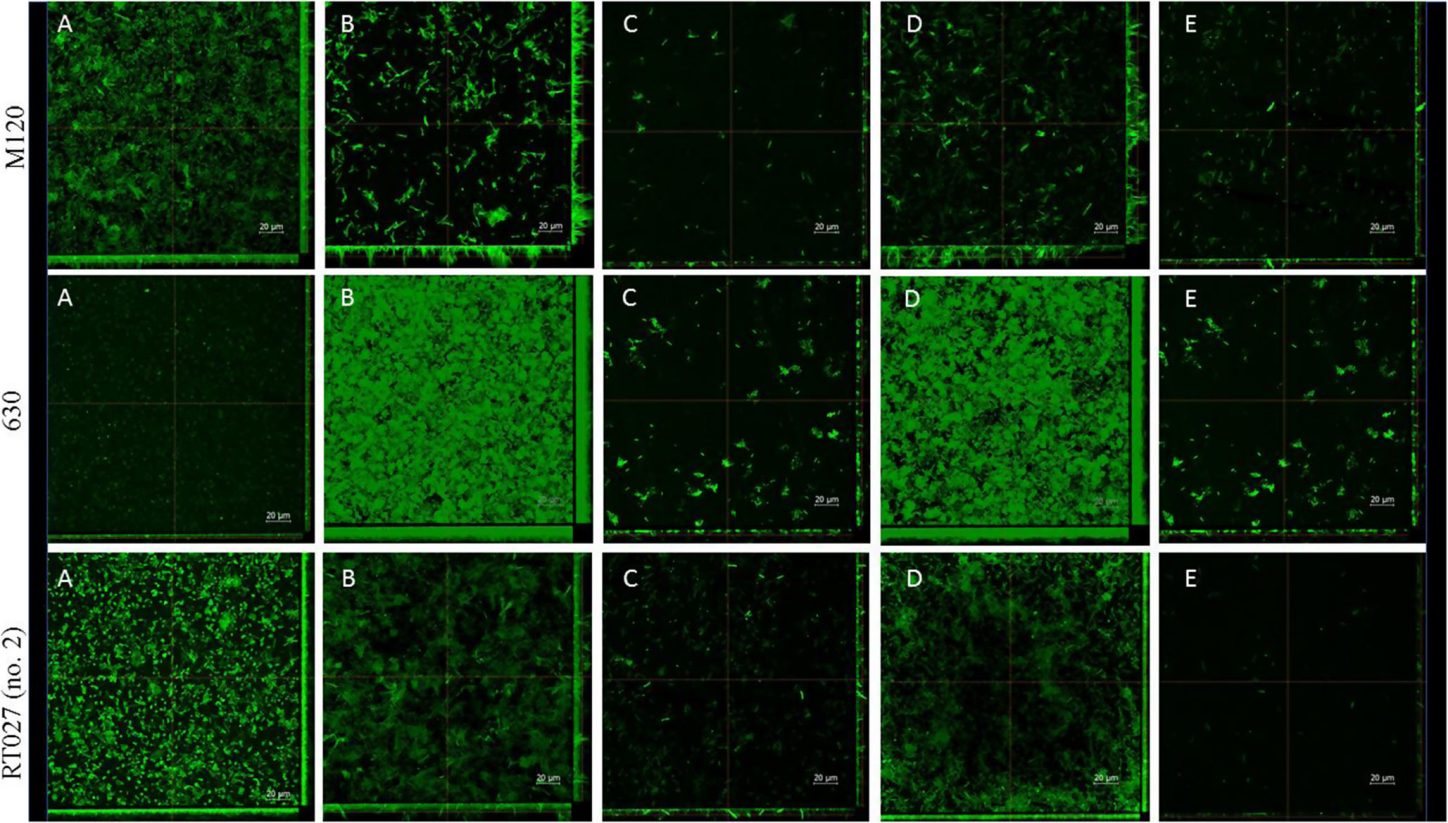
Effects of FOS and MAN on *C. difficile* biofilm formation. Representative confocal microscopy images of horizontal (xy) and vertical (xz and yz) projections of *C. difficile* biofilm structures. Vertical view was obtained using maximum intensity projection as a crosssection through marked line

To visualize the effects of FOS and MAN, two concentrations of these substances were utilized in culture with 3 different *C. difficile* strains, namely, 630, M120, and one clinical *C. difficile* RT027 that has previously been shown to produce the highest level of biofilm on titration plates. Prebiotics were used at an 8% concentration, which demonstrated strong inhibitory effects on biofilm formation by *C. difficile*, and at a sub-inhibitory (1%) concentration which was found to induce the process of biofilm formation. Images from confocal microscopy (Fig. 5.) confirmed the results from the experiment with titration plates. *C. difficile* 630 strain was found to form a thin biofilm layer in the control without prebiotics (Fig. 5. 630 A), and the other strains, M120 and RT027, produced higher amounts of biofilm in the control. The sub-inhibitory concentration (1%) of FOS and MAN (Fig. 5 B, D) acted to increase the density and roughness of *C. difficile* biofilm, most notably within strain 630 cultures. Further, the biofilm produced by strain M120 in culture with 1% FOS or MAN became rugged with holes and elongated forms (Fig. 5, M120 B and D). Alternatively, higher concentrations (8%) of prebiotics resulted in decreased biofilm biomass production with a thinner layer and a smaller number of bacterial aggregates in all tested strains compared to the control (Fig. 5 C and E).

## Discussion

*C. difficile* is the most common etiological factor for nosocomial diarrhoea. The increase in CDI incidence has been caused by emergence of hyperepidemic strains, especially those belonging to the genotype NAP1/B1/RT027. These strains often exhibit multi-drug resistance and highly expressed virulence factors, such as higher toxin production and high spore resistance to adverse conditions [6]. Furthermore, a study performed by Collins et al. reported that treatment with the disaccharide trehalose acted to enhance pathogenic *C. difficile* virulence [24].

In the current study, we examined the effects of specific carbohydrates prebiotics and candidate prebiotics: CEL, FOS, INU, MAN, and RAF on various *C. difficile* strains. We focused primarily on clinical strains characterized as PCR-ribotype 027 due to their hyper-pathogenic characteristics. We, specifically, investigated how non-digestible prebiotics affect in vitro adhesion of *C. difficile*, which is the initial step of colonization and biofilm formation.

FOS and MAN exhibited the strongest anti-adhesion potential in all the 3 human epithelial cell lines. Hartman et al. reported on the anti-adhesive potential of mannosides on *E. coli.* They found that mannose and mannans decreased *E. coli* adhesion to HT-29 cells by up to 90% [25]. Further, Shoaf et al. employed FOS to reduce adherence of enteropathogenic *E. coli* (EPEC) to Caco-2 cells and reported approximately 40% inhibition of adherence [26]. Altamimi et al. also investigated the effect of different oligosaccharides, including raffinose and cellobiose on gut bacteria, including *C. difficile*. However, no significant effects were observed in this study. Other carbohydrates (chitooligose, lactulose, stachyose) have also not exhibited significant anti-adhesive properties in HT-29 cell lines (non-mucus and mucus secreting) [22]. Similarly, in our study, CEL and INU demonstrated the largest CFU median in all the 3 cell lines and promoted adhesion in only a few bacterial strains (data not shown), while RAF was found to only significantly inhibit adhesion of *C. difficile* in HT-29 cells. Alternatively, Wang et al. reported on the effect of carbohydrates on adhesion of intestinal pathogens to HT-29 cells. Mannose was found to reduce adhesion of *Vibrio cholerae* by 60% and *Campylobacter jejuni* by 30%. Moreover, FOS inhibited adhesion of *Salmonella* Typhimurium by 71.4%. However, these saccharides did not effectively inhibit adhesion of *E. coli* [27]. The mechanisms responsible for inhibiting and promoting adhesion are not clearly understood. However, sugars have been shown to function as adhesion inhibitors and affect the expression of surface proteins and adhesins of bacteria. Hence, further examination of these mechanisms will be carried out in the future.

Our results suggest that specific prebiotics can affect the formation of biofilms by certain *C. difficile* strains. Media supplemented with 8% FOS and MAN functioned to statistically significantly decrease biofilm formation by *C. difficile* 630 and *C. difficile* M120. However, in the clinical strains, significantly decreased biofilm formation not observed. Interestingly, we also observed that biofilm formation was enhanced in the presence of low concentrations of prebiotics, most notably with 1% (sub-inhibitory) FOS and MAN. However, for RT027 clinical strains, observations were not statistically significant. Images from confocal laser scanning microscopy confirmed these results and allowed us to visualize the 3D architecture of biofilms. At sub-inhibitory concentration (1%) of FOS and MAN, an increase in roughness and changes in homogeneity were observed, resulting in changes from a smooth biofilm surface to a more heterologous surface and a 3D architecture containing many aggregates. The biofilm formed by *C. difficile* 630, in the absence of prebiotics, was the thinnest of the three tested strains which corroborated with results from Semenyuk et al. who described *C. difficile* 630 as a weak biofilm producer [28]. Additionally, our results showed increased biofilm formation by *C. difficile* following exposure to a sub-inhibitory concentration (1%) of FOS and MAN. Similarly, Creti et al. described strong biofilm production by wild-type *Enterococcus faecalis* after incubation with 1% MAN and fructose [29]. These results suggest that the presence of oligosaccharides in food may influence colonization and biofilm formation by bacteria in the human gastrointestinal tract.

To our knowledge, this is first study to investigate the effects of prebiotics on biofilm formation by *C. difficile* and, thus, requires further investigation*.* Powell et al. examined the effects of alginate oligosaccharide on *Pseudomonas aeruginosa* biofilm and microscopy imaging demonstrated a dose-depending reduction on biofilm formation. Moreover, alginates at 2% and 6% concentrations functioned to decrease the thickness of *P. aeruginosa* biofilm [30].

Many studies examining the adhesive properties of microorganisms employ epithelial cell lines such as HT-29 or Caco-2. We, however, used an additional mucus-secreting epithelial cell line, namely HT-29 MXT and epithelial cells from healthy human CCD 841 CoN for comparison. However, we observed no significant differences in adhesion of *C. difficile* RT027 to these 3 cell lines. These results were not in agreement with those reported by Altamimi et al. This study revealed that *C. difficile* preferred mucus secreting epithelial cells over non-mucus-secreting ones; however, only one reference strain ATCC 43255 was used in these assays [22].

Although no significant differences were observed in adherence of the clinical strains to the different cell lines, we did determine that the non-motile *C. difficile* M120 strain adhered more strongly than the motile *C. difficile* 630 or RT027 strains. The M120 strain has deletion of the entire F3 region which is responsible for encoding flagellin [31]. However, mice experiments employing the 630 strain with other flagellar mutants revealed that flagella are not required for adherence and colonization by this *C. difficile* strain [32]. Thus, higher adhesion of non-motile strains may be simply explained by more rapid settling of the bacterial cells onto the epithelia compared to that by motile strains.

## Conclusion

Specific prebiotics exhibit anti-adhesive properties and are safe and ecologically friendly. FOS and MAN possessed the highest anti-adhesive activity against *C. difficile* strains, thereby leading to decreased biofilm formation. For clinical strains, the effect was observable; however, values were not statistically significant. Importantly, we also determined that sub-inhibitory concentrations of FOS and MAN serve to enhance biofilm formation, which is better visualized using confocal microscopy imaging than crystal violet staining. To our knowledge, this is the first report to address the effects of prebiotics and candidate prebiotics on adhesion and biofilm formation of hypervirulent RT027 *C. difficile* strains.
